# Health-related quality of life and its influencing factors for patients with hypertension: evidence from the urban and rural areas of Shaanxi Province, China

**DOI:** 10.1186/s12913-016-1536-x

**Published:** 2016-07-18

**Authors:** Yulian Zhang, Zhongliang Zhou, Jianmin Gao, Dan Wang, Qian Zhang, Zhiying Zhou, Min Su, Dan Li

**Affiliations:** School of Public Health, Health Science Center, Xi’an Jiaotong University, Xi’an, China; School of Public Policy and Administration, Xi’an Jiaotong University, Xi’an, China; Nursing Department, Shaanxi Provincial People’s Hospital, Xi’an, China

**Keywords:** Hypertension, HRQoL, EQ-5D, Tobit regression model

## Abstract

**Background:**

Hypertension is an important public health issue in China, but there are few studies on health-related quality of life (HRQoL) for patients with hypertension in China. This study aims to examine the HRQoL as measured by EQ-5D and investigate the factors that influence HRQoL for patients with hypertension in Shaanxi Province, China.

**Methods:**

Data were collected from the Shaanxi’s fifth National Health Service Survey conducted in 2013. EQ-5D was employed to measure the HRQoL for patients with hypertension. The Chinese population-based preference trade-off time (TTO) model was used to convert the EQ-5D values. All descriptive analyses, including demographic characteristics, socio-economic status and clinical characteristics, were stratified by urban and rural residence. Tobit regression model was used to investigate the influencing factors of HRQoL.

**Results:**

A statistically significant difference was observed between the EQ-5D utility scores of urban (0.891) and rural hypertension patients (0.870). The urban hypertension patients showed significantly higher utility scores than the rural patients in three of the five dimensions, namely usual activities, pain / discomfort and anxiety / depression. The influencing factors of HRQoL for hypertension patients in China included age, marital status, education level, employment status, physical activity and medical examination. For patients aged 55 years and above, EQ-5D utility score decreased significantly with increasing age. The EQ-5D score increased with higher education level. Married patients showed a higher EQ-5D score than divorced and widowed patients, and employed patients showed a higher score than unemployed and retired patients. Regular physical activity and medical examination had a positive effect on the HRQoL of hypertension patients.

**Conclusions:**

Our study indicated that urban hypertension patients might have higher HRQoL than rural patients in Shaanxi, China. To enhance HRQoL, it is necessary to strengthen the health education for hypertension patients to improve hypertension prevention and to adopt healthy habits such as regular physical activity. It is also important to strengthen the management and monitoring of hypertension in the elderly, and further implement the free medical examination program for the elderly under the public health programs.

**Electronic supplementary material:**

The online version of this article (doi:10.1186/s12913-016-1536-x) contains supplementary material, which is available to authorized users.

## Background

Hypertension is an important public health issue worldwide due to its high prevalence and mortality rate [1, 2]. The world health statistics 2012 [3] reported the growing burden of noncommunicable diseases, in which one third of adults suffered from hypertension, and half of the total death toll caused by stroke and heart disease is due to hypertension. The Chinese cardiovascular disease report 2013 [4] published in 2014 reported that the prevalence rate of hypertension in China was rising. In the year of 2012, the prevalence of hypertension was 24 % in Chinese adults over 15 years old, and 270 million (at least two of every 10 adults) suffered from hypertension in China. From 1979 to 2002, the prevalence of hypertension increased dramatically in rural area and the difference in the incidence of hypertension between urban and rural populations was narrowing. Hypertension has become one of the major public health issues in both urban and rural areas in China.

Studies have indicated that hypertension seriously affects patients’ health. Saleem et al. [5] studied the health-related quality of life (HRQoL) profile of hypertension population in Pakistan with EuroQol questionnaire (EQ-5D), and found that hypertension has an adverse effect on patients’ well-being and HRQoL. A study by Mena-Martin et al. also demonstrated that patients with known hypertension present a poorer HRQoL [6]. This is consistent with other literature reports on HRQoL of hypertension patients in other countries [7]. Studies of hypertension patients in China have mainly focused on the prevalence and influencing factors of hypertension. Chen et al. studied the prevalence, awareness and other aspects associated with hypertension in Yi ethnic population in Shilin County of Yunnan, China [8]. They reported the risk factors for hypertension included older age, smoking, alcohol consumption, family history of high blood pressure, overweight and obesity, and protective factors such as being slim and with higher education. However, few studies have focused on the HRQoL of hypertension population in China. Wang et al. examined the relationship between hypertension and HRQoL in a general population in Shanghai, China using the Mandarin version of 36-item Short Form (SF-36) [9]. Nevertheless, to date, no report has been published concerning HRQoL of hypertension patients in China as measured by EQ-5D.

HRQoL is an assessment of health state based on modern concept of healthcare, which reflects the physical, psychological, social and emotional well beings of patients [10]. It takes into account not only the disease itself, but also the psychological and social impact of the disease [11]. EQ-5D is a generic preference-based tool developed by the EuroQol Group in 1990 for subjectively describing and valuing HRQoL [12, 13]. EQ-5D-3 L (EQ-5D 3 level version, hereafter referred to as the EQ-5D), one of the four major measurement systems of HRQoL [14], has been widely used in China in recent years [15, 16]. Meanwhile, its validity and reliability have been demonstrated in mainland China [17]. HRQoL reflects the health utility index score; however, health utility scores cannot be calculated directly from the measurement results by EQ-5D. Therefore, a population-based preference trade-off time (TTO) model is required to convert the measurements into health utility scores of the population. Recently, Liu et al. has developed a Chinese general population-based value set for EQ-5D health states [18], which is capable of converting health states measured by the EQ-5D-3 L descriptive system to utility scores.

In this study, we aim to examine the health status of hypertension patients in urban and rural areas of Shaanxi Province, China using EQ-5D as a measure of HRQoL, and to investigate the influencing factors of HRQoL. It is the first attempt to use the Chinese population-based preference value set for EQ-5D to measure HRQoL of hypertension patients, which provides a reference for further research on the health status of hypertension patients.

## Methods

### Data source

Data were collected from the Shaanxi’s fifth National Health Service Survey (NHSS). NHSS is organized and directed by the Center of Health Statistics and Information under the Ministry of Health of China and has been conducted every five years since 1993. In the Shaanxi’s NHSS 2013, the stratified multistage random cluster sampling method was employed. In the first stage, 32 counties/districts were selected randomly in Shaanxi Province. In the second stage, 160 townships were selected in sampled counties/districts. In the third stage, 320 villages/communities were selected in sampled townships. In the last stage, 60 households were randomly selected in each sampled village/community and the selected households that could not be accessed were replaced by their neighbors. Finally, 20,700 households were surveyed.

Considerable quality control measures were implemented during the process of data collection. Before the interviews, well-trained investigators would explain the purposes and confidentiality of the survey to interviewees. If there are missing information and errors in the questionnaires, re-survey would be required the next day after checking the completeness of questionnaires by the survey manager at the end of each day. Moreover, 5 % households from the whole samples were resurveyed to examine the survey quality and the concordance rate between the first survey and resurvey was 95 %. Myer’s index was employed to test the representativeness of the survey. It showed that the sample was representative and did not have age preference comparing with the general population (Myer’s index equals 2.16).

The NHSS questionnaire mainly includes general family and each member conditions, self-report health status in multiple dimensions and health-related behaviors (smoking, alcohol consumption, and physical activity). A face-to-face interview was conducted for each household in the residents’ home. Residents were required to answer questions themselves, but if residents were not at home or unable to answer at the time of the survey, other family members could serve as proxy. Overall, 69.13 % selected residents answered questions themselves and the questionnaires for other residents were answered by their family members instead.

All hypertension patients were identified by the question “Have you been diagnosed with hypertension by a doctor?”. The purpose of this study dictated that only the individuals with hypertension aged above 15 years old were included in this study. Consequently, the sample size of this study was 6145, of which 2424 were from the urban area (urban group) and 3721 were from the rural area (rural group).

### Measurements of EQ-5D utility score

HRQoL measured by EQ-5D was used as the health outcome measure in this study. The EQ-5D-3 L consists of five dimensions: mobility, self-care, usual activities, pain/discomfort, and anxiety/depression. Each dimension has three levels of response or severity (no problems, moderate problems and extreme problems). A total of 243 possible health states can be defined by combining one level from each of the five dimensions [19]. The Chinese utility value set (Table 1) was used to obtain utility scores for EQ-5D health states [18]. C is a constant. MO2, SC2, UA2, PD2 and AD2 terms are 0 for the absence of level 2 problems in mobility, self-care, usual activities, pain/discomfort and anxiety/depression, respectively. MO3, SC3, UA3, PD3 and AD3 are 0 for the absence of level 3 problems in those dimensions. N3 was 1 if any level 3 problems were present in a state and 0 if otherwise. Thus, the utility scores can be calculated for all of the 243 health states based on Table 1. For example, the utility score for “23221” was U = 1 - (0.039 + 0.099 + 0.208 + 0.074 + 0.092 + 0 + 0.022) = 0.466. The utility score for state 11111 (no problems on any of the five dimensions) was 1, and the score for state 33333 (the worst health state) was -0.149. Consequently, the range of Chinese utility values was from -0.149 to 1.

**Table 1:** The Chinese utility values for EQ-5D health states

C	MO2	MO3	SC2	SC3	UA2	UA3	PD2	PD3	AD2	AD3	N3
0.039	0.099	0.246	0.105	0.208	0.074	0.193	0.092	0.236	0.086	0.205	0.022

### Statistical analysis

#### Descriptive statistics

Descriptive statistics were calculated for the basic demographic variables of HRQoL. Mean and standard deviations (SD) were calculated for continuous variables, and frequencies and percentages were calculated for categorical variables. 95 % confidence interval (CI) was used to show the difference in the EQ-5D utility scores between the urban and rural groups. Chi-square test was used to compare the difference in constituent ratio in each dimension of EQ-5D between the urban and rural groups. One-way analysis of variance (ANOVA) was used to test the difference in EQ-5D utility scores at different levels of each variable. All statistical analyses were performed using STATA version 12.0 for Windows.

#### Tobit regression model

EQ-5D utility score is a censored variable, i.e. a large proportion of respondents had the health utility score of 1. Therefore, the Tobit regression model suitable for censored or bounded data was used to analyze the influencing factors of EQ-5D utility score of the urban and rural groups [10, 20, 21]. Three Tobit regression models were employed by using the data of urban group, rural group, and both of them, in which the EQ-5D utility scores were the dependent variables. The independent variables included area, the natural logarithm of per capita consumption expenditure, gender, age, ethnic group, region, marital status, education level, employment status, health insurance coverage, smoking status, drinking status, physical activity, health education, and medical examination.

Area included urban area and rural area. Per capita consumption expenditure referred to the net expenditure after deducting medical and health spending from the total expenditure per year calculated based on household population. Ethnic groups included Han and others (Hui, Tibetan, Mongolia, etc). Regions were divided into Shannan (south of Shaanxi), Guanzhong (central of Shaanxi) and Shanbei (north of Shaanxi). Marital status indicates whether the respondent is married, single, divorced or widowed. Education level includes illiterate, primary school, junior high school, senior high/technical school, and college and above. Employment status indicates whether the respondent is employed, retired or unemployed. Medical insurance includes urban resident medical insurance, urban employee medical insurance, new rural cooperative medical system, commercial medical insurance and other medical insurance. The medical insurance information was derived by asking questions like “Are you covered by any medical insurance?”. For the variables of smoking status, drinking status, physical activity, health education and health examination, the respondents answered “yes” or “no” to the questions like “Do you smoke now?”, “Have you drunk alcohol during the last 12 months?”, “Do you take physical exercise every week?”, “Have you been educated by medical workers to prevent and cure chronic disease during last 3 months” and “Have you undergone medical examination during the last 12 months?”. All the categorical variables in this model were set as dummy variables.

## Results

### Distribution of the EQ-5D self-classified health states

As shown in Table 2, the highest proportion of hypertension patients reported moderate and extreme problems in the pain / discomfort dimension of EQ-5D, with 28.97 % of the urban group and 35.35 % of the rural group, respectively. In contrast, the lowest proportion of patients reported problems in the self-care dimension, with 9.43 % of the urban group and 12.5 % of the rural group, respectively. Overall, a significant higher proportion of the rural group reported problems in the mobility, usual activities, pain/discomfort, and anxiety/depression dimensions, compared with the urban hypertension population (*P* < 0.05).

**Table 2:** Distribution of the EQ-5D self-classified health states of the urban and rural population with hypertension in Shaanxi Province, China

	Urban hypertensive population (%)			Rural hypertensive population (%)			*P* value*
	No problem	Moderate problem	Extreme problem	No problem	Moderate problem	Extreme problem	
Mobility	81.72	16.25	2.02	79.27	18.55	2.18	0.049
Self-care	88.57	8.79	2.64	87.50	9.92	2.58	0.331
Usual activities	84.94	11.47	3.59	81.40	14.03	4.57	0.002
Pain/discomfort	71.03	26.70	2.27	64.65	32.61	2.74	<0.001
Anxiety/depression	86.37	12.02	1.61	81.29	17.45	1.26	<0.001

* Chi-square test was employed to test the difference in constituent ratio in each dimension of EQ-5D between the urban and rural groups. Missing data: the numbers of respondents with missing data are 0, 1, 0, 1, 3 in urban area and 2, 1, 1, 1, 1 in rural area for the variables of mobility, self-care, usual activities, pain/discomfort, anxiety/depression are respectively

### EQ-5 utility scores

Table 3 shows the utility scores of EQ-5D and its each dimension of the urban and rural groups calculated based on the Chinese value set for EQ-5D health states. A statistically significant difference was observed in the EQ-5D utility scores between the urban (0.891) and rural groups (0.870). Moreover, the utility scores of the urban group were higher than those of the rural group in three of the five dimensions, namely usual activities, pain/discomfort and anxiety/depression, which were statistically significant. There was no significant difference in utility scores in the mobility and self-care dimension between the urban and rural groups.

**Table 3:** Utility scores of EQ-5D and its each dimension of the urban and rural groups

Dimensions	Urban group (95 % CI)	Rural group (95 % CI)
Mobility	−0.021 (−0.023, −0.019)	−0.025(−0.025, −0.022)
Self-care	−0.015(−0.016, −0.013)	−0.016(−0.017, −0.014)
Usual activities	−0.015(−0.017, −0.014)	−0.019(−0.021, −0.018)
Pain/discomfort	−0.030(−0.032, −0.028)	−0.037(−0.038, −0.035)
Anxiety/depression	−0.014(−0.015, −0.012)	−0.018(−0.019, −0.016)
EQ-5D utility	0.891(0.883, 0.899)	0.870(0.863, 0.876)

Table 4 shows the EQ-5D scores of each categorical variable. For both urban and rural groups, the EQ-5D utility scores of males were significantly higher than those of females (*P* < 0.05). For different age groups, the difference of scores was statistically significant. The scores for the groups with older age were lower than that for the groups with younger age in both urban and rural area. The scores were lower for hypertension patients living in Shanbei than patients living in Shannan and Guanzhong. Divorced patients, illiterate patients and unemployed patients scored the lowest under their respective category. Non-smokers and non-drinkers had higher scores than smokers and drinkers, respectively. Patients with physical activity had higher scores than those without it.

**Table 4:** Univariate analysis of the EQ-5D utility scores and categorical variables

	Urban group			Rural group		
	Mean	SD	*P* value*	Mean	SD	*P* value*
Gender						<0.001
Male	0.906	0.181	<0.001	0.887	0.192	
Female	0.878	0.214		0.858	0.218	
Age Group						
35–44	0.959	0.120		0.946	.118	
45–54	0.948	0.130	<0.001	0.929	0.143	<0.001
55–64	0.917	0.169		0.893	0.183	
≥ 65	0.845	0.233		0.803	0.250	
Ethnic group Han	0.890	0.201	0.670	0.870	0.207	0.463
Others	0.910	0.251		0.832	0.232	
Region						
Shannan	0.875	0.207	<0.001	0.879	0.197	0.015
Guanzhong	0.899	0.214		0.869	0.210	
Shanbei	0.878	0.195		0.850	0.215	
Marital status			<0.001			
Married	0.908	0.184		0.887	0.196	<0.001
Single	0.915	0.174		0.846	0.216	
Divorced	0.797	0.257		0.795	0.243	
Widowed	0.886	0.149		0.980	0.600	
Education level			<0.001			<0.001
Illiterate	0.797	0.263		0.815	0.241	
Primary school	0.874	0.202		0.877	0.201	
Junior high school	0.919	0.174		0.921	0.155	
Senior high / technical school	0.928	0.162		0.922	0.163	
College and above	0.948	0.123		0.995	0.026	
Employment status			<0.001			<0.001
Employed	0.937	0.132		0.905	0.169	
Retired	0.890	0.205		0.863	0.217	
Unemployed	0.823	0.251		0.778	0.265	
Insurance coverage						
Uninsured	0.890	0.201	0.071	0.833	0.174	0.175
Insured	0.931	0.177		0.870	0.209	
Smoking status			<0.001			<0.001
Yes	0.882	0.210		0.860	0.219	
No	0.923	0.153		0.900	0.167	
Drinking status			<0.001			<0.001
Yes	0.880	0.209		0.863	0.214	
No	0.947	0.124		0.930	0.140	
Physical activity			<0.001			
Yes	0.929	0.139		0.892	0.170	0.078
No	0.854	0.239		0.865	0.216	
Health education						
Yes	0.891	0.192	0.123	0.871	0.207	0.107
No	0.890	0.215		0.858	0.218	
Health examination						
Yes	0.899	0.175	0.924	0.870	0.200	0.275
No	0.889	0.231		0.869	0.213	

* ANOVA was employed to test the difference in EQ-5D utility scores at different levels of each variable. Missing data for the variables: marital status (urban/rural: 1/2), employment status (urban/rural: 5/0), insurance coverage (urban/rural: 8/2), smoking status (urban/rural: 2/0), drinking status (urban/rural: 24/35), physical activity (urban/rural: 17/12), health education (urban/rural: 0/4) and health examination (urban/rural: 1/8)

### Influencing factors of HRQoL

Table 5 shows the description of the independent variables in Tobit regression models. The partial regression coefficients obtained by Tobit regression model are shown in Table 6. From model 1, the partial regression coefficient of urban patients was 0.011 (*p* < 0.05), which suggested that, with other factors controlled, the EQ-5D score of the urban patients was 0.011 higher than the rural patients and the difference was statistically significant. From model 2, results indicated that the influencing factors of HRQoL for the urban patients included age, marital status, education level, employment status, physical activity and medical examination. In the urban area, with other factors controlled, the EQ-5D score of the age groups “55-64” and “≥65” were 0.037 and 0.074 lower than the age group “15-34”, respectively. The married patients had higher scores than divorced patients and widowed patients, and the EQ-5D score increased with higher education level. Employed patients showed a higher score than unemployed and retired patients. The EQ-5D scores of patients with physical activity and medical examination are 0.053 and 0.022 higher than those without it, respectively. For model 3, the influencing factors of HRQoL for the rural patients included age, region, marital status, education level, employment status, and medical examination. In the rural area, with other factors controlled, the EQ-5D score of the age groups “55-64” and “≥65” were 0.046 and 0.099 lower than the age group “15-34”, respectively. Patients in Shannan area showed higher scores than those in Shanbei, and married patients had higher scores than divorced and widowed patients. The EQ-5D score increased with higher education level, and employed patients had a higher score than unemployed patients. The EQ-5D score of patients with regular medical examination was 0.026 higher than those without it.

**Table 5:** Description of independent variables

Variables	Urban and rural *N* (%)	Urban group *N* (%)	Rural group *N* (%)
Area			
Urban	2,424 (39.5)	—	—
Rural^a^	3,721 (60.5)	—	—
Ln of expenditure (RMB)	8.60 ± 0.79	8.86 ± 0.74	8.44 ± 0.78
Gender			
Male^a^	2,629 (42.8)	1,124 (46.4)	1,505 (40.5)
Female	3,516 (57.2)	1,300 (53.6)	2,216 (59.5)
Age Group(years)			
15–44^a^	410 (6.7)	155 (6.4)	255 (6.8)
45–54	1,218 (19.8)	415 (17.1)	803 (21.6)
55–64	1,918 (31.2)	694 (28.6)	1,124 (32.9)
≥ 65	2,599 (42.3)	1,160 (47.9)	1,439 (38.7)
Ethnic group			
Han^a^	6,043 (98.3)	2,403 (99.1)	3,640 (97.8)
Others	102 (1.7)	21 (0.9)	81 (2.2)
Region			
Shannan^a^	1,980 (32.2)	601 (24.8)	1379 (37.1)
Guanzhong	3,328 (54.2)	1535 (63.3)	1793 (48.2)
Shanbei	837 (13.6)	288 (11.9)	549 (14.7)
Marital status			
Married^a^	4,946 (80.5)	2,004 (82.7)	2,942 (79.1)
Single	96 (1.6)	23 (0.9)	73 (1.9)
Divorced	1,059 (17.2)	372 (15.4)	687 (18.5)
Widowed	41 (0.7)	24 (1.0)	17 (0.5)
Education level			
Illiterate^a^	1,718 (28.0)	420 (17.3)	1,298 (34.9)
Primary school	1,886 (30.7)	615 (25.4)	1,271 (34.1)
Junior high school	1,598 (26.0)	705 (29.1)	893 (24.0)
Senior high / technical school	720 (11.7)	486 (20.0)	234 (6.3)
College and above	223 (3.6)	198 (8.2)	25 (0.7)
Employment status			
Employed^a^	3,522 (57.4)	916 (37.9)	2,606 (70.0)
Retired	1,000 (16.3)	885 (36.6)	115 (3.1)
Unemployed	1,618 (26.4)	618 (25.5)	1,000 (26.9)
Insurance coverage			
Uninsured^a^	54 (0.9)	43 (1.8)	16 (0.4)
Insured	6,081 (99.1)	2,381 (98.2)	3,705 (99.6)
Smoking status			
Yes^a^	1,432 (23.3)	546 (22.5)	887 (23.8)
No	4,711 (76.7)	1,878 (77.5)	2,834 (76.2)
Drinking status			
Yes^a^	777 (12.8)	398 (16.4)	397 (10.7)
No	5,309 (87.2)	2,026 (83.6)	3,324 (89.3)
Physical activity			
Yes	1,828 (29.9)	1,175 (48.5)	668 (18.0)
No^a^	4,288 (70.1)	1,249 (51.5)	3,053 (82.0)
Health education			
Yes	4,723 (76.9)	1,607 (66.3)	3,119 (83.8)
No^a^	1,418 (23.1)	817 (33.7)	602 (16.2)
Health examination			
Yes	3,317 (54.1)	1,423 (58.7)	1,898 (51.0)
No^a^	2,819 (45.9)	1,001 (41.3)	1,823 (49.0)

^a^ Reference variables in the Tobit regression model. Ln of expenditure means natural logarithm of per capita consumption expenditure

**Table 6:** Influencing factors of EQ-5D utility scores from Tobit regression model

	Urban and Rural (model 1)		Urban (model 2)		Rural (model 3)	
	dy/dx	SE	dy/dx	SE	dy/dx	SE
Area (Rural)						
Urban	0.011**	0.006	—	—	—	—
Lnperexp^a^	0.007	0.003	0.007	0.006	0.006	0.004
Gender (Male)						
Female	0.007	0.006	0.014	0.009	0.001	0.008
Age group (15–34)						
45–54	−0.019	0.012	−0.013	0.020	−0.023	0.015
55–64	−0.042***	0.012	−0.037**	0.019	−0.046***	0.015
≥ .0	−0.088***	0.012	−0.074***	0.020	−0.099***	0.016
Ethnic group (Han)						
Others	−0.012	0.018	−0.010	0.038	−0.019	0.021
Region (Shannan)						
Guanzhong	0.003	0.006	0.013	0.009	−0.001	0.007
Shanbei	−0.024***	0.008	−0.013	0.013	−0.031***	0.009
Marital status (Married)						
Single	−0.018	0.019	0.011	0.041	−0.028	0.022
Divorced	−0.027***	0.007	−0.037***	0.010	−0.019**	0.008
Widowed	−0.090**	0.031	−0.070*	0.036	−0.110**	0.062
Education (Illiterate)						
Primary school	0.025***	0.006	0.029**	0.011	0.023***	0.008
Junior high school	0.052***	0.008	0.058***	0.012	0.048***	0.010
Senior high / technical school	0.052***	0.010	0.061***	0.014	0.051***	0.015
College and above	0.083***	0.018	0.069***	0.020	0.234***	0.078
Employment (Employed)						
Retired	−0.040***	0.009	−0.057***	0.011	−0.022	0.019
Unemployed	−0.058***	0.006	−0.057***	0.010	−0.060***	0.007
Insurance (Uninsured)						
Insured	−0.001	0.027	−0.031	0.032	0.066	0.047
Smoking status (Yes)						
No smoking	−0.014	0.009	−0.015	0.011	−0.013	0.009
Drinking status (Yes)						
No drinking	−0.018	0.011	−0.020	0.012	−0.016	0.012
Physical activity (No)						
Yes	0.033***	0.006	0.053***	0.008	0.011	0.008
Health education (No)						
Yes	0.003	0.006	0.001	0.008	0.009	0.008
Health examination (No)						
Yes	0.025***	0.005	0.022***	0.008	0.026***	0.007
LR	832.79		358.22		498.94	
p	<0.001		<0.001		<0.001	
N	5,879		2,241		3,629	

*P < 0.1, **P < 0.05, ***P < 0.01. N are the number of respondents included in the Logistic regression models

^a^ Natural logarithm of per capita consumption expenditure. Reference variables are in the parentheses

## Discussion

Hypertension is the most prevalent chronic non-communicable disease in China [22], which can lead to serious complications such as cerebral vascular disease, heart disease, heart failure and renal failure. With longer life expectancy in China, there has been an increasing concern on HRQoL of patients with hypertension. This is the first study that employed the EQ-5D scale and Chinese general population-based EQ-5D value set for measurement of HRQoL for adult patients (aged 15 years and above) with hypertension in urban and rural areas, which revealed the current status of HRQoL and its influencing factors for hypertension patients in China.

We found a higher proportion of hypertension patients in the rural area had moderate and extreme problems in the mobility, usual activities, pain/discomfort and anxiety/depression dimensions, as compared with patients in the urban area. This finding agreed with the utility scores of EQ-5D and its each dimension obtained for the urban and rural patients, i.e. the urban patients showed higher utility scores. Studies by Zhou et al. showed that the EQ-5D utility scores of the general population in the urban and rural areas of Shaanxi, China were 0.9569 and 0.9588, respectively. Our study revealed that the hypertension patients in Shaanxi, China had an obviously lower EQ-5D utility score than the general population. Moreover, the proportions of patients reporting moderate problems and extreme problems were also higher than those of the general population in all of the five dimensions [15, 23]. This demonstrated the negative correlation between hypertension and HRQoL. Univariate analysis of the EQ-5D utility scores and categorical variables demonstrated that male hypertension patients had a better HRQoL than female patients. HRQoL of older patients was significantly lower than younger patients, possibly because hypertension is a chronic disease which progresses with age and increasingly affects health. Education level was found to have a significant effect on the health status of hypertension patients, with higher-educated patients having a better HRQoL. There were statistically significant differences in the HRQoL among patients living in Shannan, Guanzhong and Shanbei, which may be related to the different diets in the three regions. Smoking and alcohol consumption were found to be the negative influencing factors of HRQoL, while physical activity was a positive influencing factor.

Tobit regression models further evaluated the effect of multiple factors, and demonstrated that age exerted a significant effect on the HRQoL of hypertension patients. For patients aged 55 years and older, HRQoL decreased significantly with increasing age. Marital status was one of the factors to affect the HRQoL of hypertension patients and married patients showed higher HRQoL than divorced and widowed patients, indicating that the rise of divorce rate would decrease the quality of life of hypertension patients. In both urban and rural areas, HRQoL was positively correlated with education level, and patients with higher education level showed better HRQoL. This was consistent with the findings reported by Zhou et al. [23] and Andrade et al. [24], and indicated the importance of improving the overall education level of the population. Employment status also affected the HRQoL of hypertension patients, with employed patients having a significantly better HRQoL than unemployed patients. This finding suggested that solving the employment issue and increasing the employment rate at the national level would contribute to improvement of the quality of life of hypertension patients. In addition, physical activity was a protective factor of HRQoL for urban hypertension patients, indicating that regular exercise was important for improving HRQoL of hypertension patients. In both urban and rural areas, patients who had medical examination in the past one year had a significantly better HRQoL than those who did not. Regular medical examination can not only facilitate early detection and treatment of the complications associated with hypertension, but also improve the patients’ health awareness to prevent the complications and their adoption of a healthy diet and living habit. We suggest that the health administrative departments strengthen the management and monitoring of chronic diseases in the elderly, and further implement the free medical examination program for the elderly under public health programs. Tobit regression model analysis demonstrated that hypertension patients in urban area had a higher HRQoL than those in rural area. This might be caused by the difference of education level, physical activity and medical examination between urban patients and rural patients. Compared with the rural patients, the higher education level, the more regular physical activity and medical examination were the three main reasons for the higher HRQoL of hypertension patients in urban China. Our results are consistent with other studies, demonstrating that hypertension patients in urban area had a higher HRQoL than those in rural area. For example, Pan et al [25] used the SF-26 scale and showed that the quality of life (QOL) of hypertension patients in cities was significantly higher than patients in rural area. Ma et al [26] used self-evaluation, mini-mental state examination (MMSE), activities of daily-living (ADL), and center for epidemiologic studies depression scale (CES-D) to study the QOL of older hypertension patients in the Beijing area. They found the quality of life was highly dependent on the residence area and patients in rural area showed lower QOL than patients in the urban area.

This study also has some limitations. First, although the EQ-5D utility scores showed statistically significant difference between the urban and rural hypertension patients, as the minimum clinically important difference (MCID) for the EQ-5D based on the scoring algorithm in China is not estimated yet, we cannot draw a conclusion that difference of EQ-5D utility scores is clinically important. However, as there is a significantly higher proportion of rural patients reported problems in four of five EQ-5D dimensions comparing with urban patients (see Table 2), we can reasonably make the conclusion that the urban hypertension patients might have higher HRQoL than the rural patients in Shaanxi, China, which is also supported by previous studies [25, 26]. Second, there might be some potential individual characteristics affecting HRQoL, which might cause a deviation of the results. Third, this study used cross-sectional survey data to analyze the correlation between HRQoL and the associated factors, rather than the causation. Finally, the data of this study were self-reported and might have some recall bias.

## Conclusions

We found that the urban hypertension patients might have higher HRQoL than the rural patients in Shaanxi, China. The main influencing factors of HRQoL included age, marital status, education level, employment status, health activity and medical examination. In order to improve HRQoL of hypertension patients, it is necessary to strengthen the health education for hypertension patients to improve their awareness of hypertension prevention and develop healthy living habits (such as regular physical activity). Meanwhile, the health administrative departments should strengthen the management and monitoring of hypertension in the elderly, and further implement the free medical examination program for the elderly under public health programs.

## Abbreviations

95 % CI, 95 % Confidence Interval; ANOVA, One-way Analysis of Variance; HRQoL, Health-Related Quality of Life; NHSS, National Health Service Survey; SD, Standard Deviation; TTO, Trade-Off Time

## Additional files

Additional file 1: Dataset used in this study. (XLS 971 kb) Additional file 2: Label of the variables in the dataset. (TXT 1 kb)

## References

[1] Kearney PM, Whelton M, Reynolds K, Muntner P, Wheton P, He J (2005). Global burden of hypertension: analysis of worldwide data. Lancet.

[2] Lawes CMM, Hoorn SV, Rodgers A (2008). Global burden of blood-pressure-related disease. Lancet.

[3] World Health Organization (2012). World health statistics 2012.

[4] Cardiovascular disease prevention and treatment research center, MOH, China. Chinese cardiovascular disease report 2013. Beijing: Chinese Encyclopedia Press; 2014.

[5] Saleem F, Hassali MA, Shafie AA (2014). A cross-sectional assessment of health-related quality of life (HRQoL) among hypertension patients in Pakistan. Health Expect.

[6] Mena-Martin FJ, Martin-Escudero JC, Simal-Blanco F, Carretero-Ares JL, Arzua-Mouronte D, Herreros-Fernandez V (2003). Health-related quality of life of subjects with known and unknown hypertension: results from the population-based Hortega study. J Hypertens.

[7] Poljicanin T, Ajdukovic D, Sekerija M, Pibernik-Okanovic M, Metelko Z, Vuletic Mavrinac G (2010). Diabetes mellitus and hypertension have comparable adverse effects on health-related quality of life. BMC Public Health.

[8] Chen L, Zong Y, Wei T, Sheng X, Shen W, Li J, Niu Z, Zhou H, Zhang Y, Yuan Y, Chen Q, Zhong H (2015). Prevalence, awareness, medication, control, and risk factors associated with hypertension in Yi ethnic group aged 50 years and over in rural China: the Yunnan minority eye study. BMC Public Health.

[9] Wang R, Zhao Y, He X, Ma X, Yan X, Sun Y, Liu W, Gu Z, Zhao J, He J (2009). Impact of hypertension on health-related quality of life in a population-based study in Shanghai, China. Public Health.

[10] Brazier JE, Yang Y, Tsuchiya A, Rowen DL (2010). A review of studies mapping (or cross walking) non-preference based measures of health to generic preference-based measures. Eur J Health Econ.

[11] Rabin R, Charro F (2001). EQ-5D: a measure of health status from the EuroQol Group. Ann Med.

[12] Agota S, Mark C, Nancy D. EQ-5D Value Sets Inventory. Comparative Review and User Guide (POD). 2007:13-15.

[13] Subramaniam M, Abdin E, Poon LY, Vaingankar JA, Lee H, Chong SA, Verma S (2014). EQ-5D as a measure of programme outcome: Results from the Singapore early psychosis intervention programme. Psychiatry Res.

[14] Wu Y, Liu K, Tang X, Cao Y, Wang J, Li N (2012). Empirical research of measuring elderly health utility in the outskirts of Beijing by using European quality of life 5-dimensions. Beijing Da Xue Xue Bao.

[15] Tan Z, Liang Y, Liu S (2013). Health-related quality of life as measured with EQ-5D among populations with and without specific chronic conditions: a population-based survey in Shaanxi Province, China. PLoS One.

[16] Sun S, Chen J, Johannesson M (2011). Regional differences in health status in China: population health-related quality of life results from the National Health Services Survey 2008. Health Place.

[17] Wang H, Patrick D, Edwards T, Skalicky A, Zeng H, Gu W (2012). Validation of the EQ-5D in a general population sample in urban China. Qual Life Res.

[18] Liu G, Wu H, Li M (2014). Chinese Time Trade-Off Values for EQ-5D Health States. Value Health.

[19] Brooks R (1996). EuroQol: the current state of play. Health Policy.

[20] Sullivan PW, Ghushchyan V (2006). Mapping the EQ-5D Index from the SF-12: US General Population Preferences in a Nationally Representative Sample. Med Decis Mak.

[21] Diels J, Hamberg P, Ford D (2015). Mapping FACT-P to EQ-5D in a large cross-sectional study of metastatic castration-resistant prostate cancer patients. Qual Life Res.

[22] Center for Health Statistics and Information, MOH, China. An Analysis Report of National Health Services Survey in China. Volume 2009. Peking Union Medical College Press; 2008.

[23] Zhou Z, Zhou Z, Li D, Wang D, Shen C, Fang Y (2015). Analyzing the health-related quality of life of urban and rural residents in Shaanxi: estimation based on the EQ-5D value sets. Chinese Health Econ.

[24] Andrade JM, Rios LR, Teixeira LS, Vieira FS, Mendes DC, Vieira MA, Silveira MF (2014). Influence of socioeconomic factors on the quality of life of elderly hypertension individuals. Cien Saude Colet.

[25] Pan Y, Ye Y, Zhu J, Gong H (2014). Analysis of influencing factors on quality of life (QOL) of patients with hypertension by SF-36 scale. Fudan Univ J Med Sci.

[26] Ma L, Tang Z, Guan S (2008). Study on quality of life of elderly hypertension patients in Beijing. Chin J Geriatr Heart Brain Vessel Dis.

